# Spontaneous Pneumothorax With Subcutaneous Emphysema: A Rare Complication of Respiratory Syncytial Virus Infection

**DOI:** 10.14740/jocmr2353w

**Published:** 2016-01-26

**Authors:** Carmen Silva, Ana Filipe Almeida, Catarina Ferraz, Teresa Nunes, Luisa Guedes Vaz

**Affiliations:** a Pediatric Department, Hospital Pediatrico Integrado, Centro Hospitalar Sao Joao, Porto, Portugal; b Pediatric Pulmonology Unit, Hospital Pediatrico Integrado, Centro Hospitalar Sao Joao, Porto, Portugal

**Keywords:** Spontaneous pneumothorax, Bronchiolitis, Respiratory syncytial virus infection

## Abstract

Viral bronchiolitis is the most common lower respiratory tract infection in infants and children under the age of 2. Respiratory syncytial virus (RSV) is the infecting agent in more than 50% of the cases. Usually the clinical course is uneventful and complications are uncommon. Secondary air leaks are a recognized rare complication of bronchiolitis, although the real incidence remains unknown. We report a case of a 21-month-old female that developed a spontaneous pneumothorax (PNO) with subcutaneous emphysema (SE) late in the course of RSV acute bronchiolitis. Additional investigation ruled out any underlying disease predisposing to spontaneous PNO. Physicians, especially those who work with small children, must be aware of this uncommon complication of bronchiolitis that may appear late in the course of the disease despite an initial clinical improvement.

## Introduction

Bronchiolitis, defined as an inflammation of the bronchioles, is usually caused by an acute viral infection. Viral bronchiolitis is the most common lower respiratory tract infection in infants and children under the age of 2 [1]. The most commonly identified infectious agent is the respiratory syncytial virus (RSV), identified in more than 50% of the cases. Other pathogens, in descending order of frequency, are rhinovirus, adenovirus, metapneumovirus, influenza and parainfluenza virus, enterovirus and bocavirus [2]. Although the clinical course is usually benign, a small number of patients may develop complications. Children at highest risk of severe disease and complications include those born prematurely, young infants (under 3 months) and those with a history of chronic lung disease of infancy (CLDI), hemodynamically significant congenital heart disease (especially conditions that cause pulmonary hypertension) and neuromuscular diseases or immunodeficiencies [3-5]. Unfrequent complications of this common illness should be well known to avoid delays in recognition and treatment.

We report a case of a child who developed a spontaneous pneumothorax (PNO) with subcutaneous emphysema (SE) late in the course of RSV acute bronchiolitis (on the seventh day of treatment) during the winter of 2015, and a review of the literature.

## Case Report

**Figure 1:**
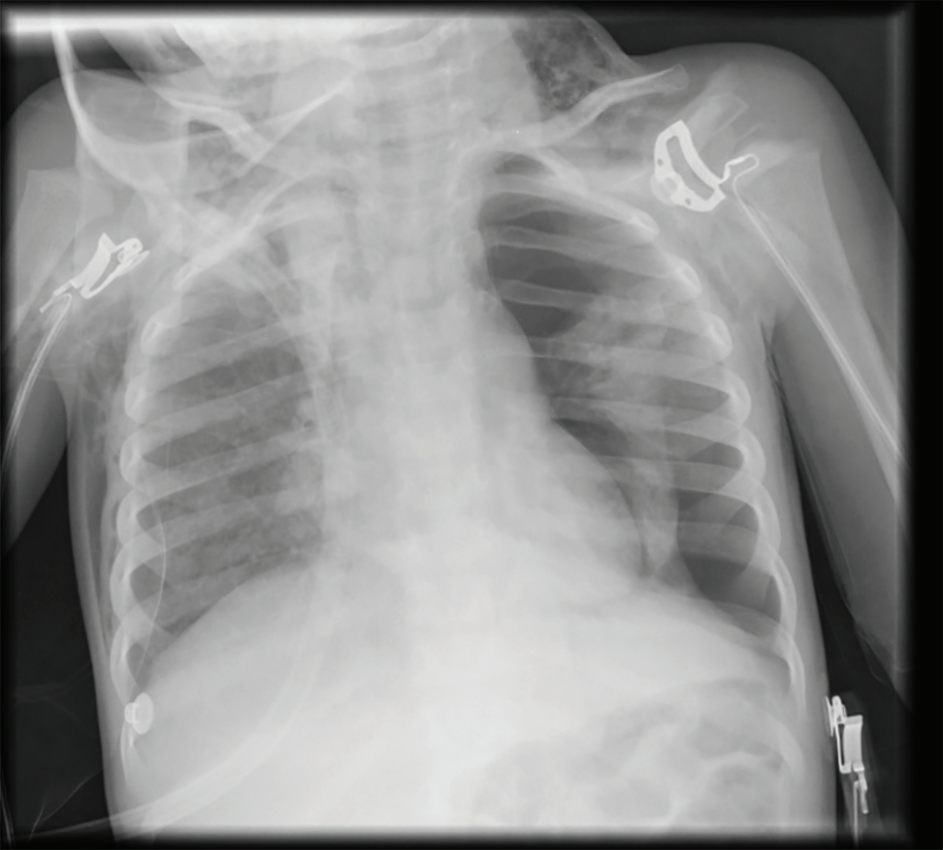
Chest X-ray with left-sided pneumothorax and cervical subcutaneous emphysema.

A 21-month-old female was admitted to hospital with the diagnosis of acute bronchiolitis with hypoxemia. She had a history of 35 weeks prematurity, slight pulmonary valve stenosis and failure to thrive; vaccination schedule was accomplished, including pneumococcal coverage. Her mother was asthmatic. Oxygen supplementation, inhaled bronchodilators and prednisolone were started. After an initial period of clinical improvement, on the third day of hospital stay, she had resurgence of fever and worsening of respiratory distress. She had a chest X-ray and laboratory tests without significant changes. RSV was identified in nasal and pharyngeal secretions. Two days later she had no fever; however, there was progressive worsening of the respiratory distress and on the seventh day of hospital stay, she developed swelling of the neck and subclavian area corresponding to SE. Chest X-ray revealed a left large volume PNO (Fig. 1), not hypertensive, and she was transferred to the pediatric pulmonology unit of our hospital (tertiary). A chest drain was inserted with aspiration and high flow oxygen was started, with progressive PNO and SE resolution. The patient was discharged after 6 more days of hospitalization. At 1-month reassessment, the patient was clinically well with slight asymmetry of breath sounds in the upper third of the left hemithorax. Additional investigation ruled out any underlying disease predisposing to spontaneous PNO such as α1-antitrypsin deficiency, cystic fibrosis (normal sweat test), immunodeficiency or underlying parenchymal lung disease (normal high-resolution computer tomography). Also, cardiac evaluation did not reveal any clinical worsening.

## Discussion

In most cases, the clinical course of bronchiolitis is uneventful and complications are uncommon. Secondary air leaks are a recognized rare complication of bronchiolitis, although the incidence remains largely unknown and limited to a few case reports described in the literature [6-15].

Pathophysiologically, the main mechanisms that lead to spontaneous PNO are rupture of alveolar bleb (congenital or acquired), destructive parenchymal disease and alveolar rupture secondary to proximal airway obstruction. In bronchiolitis, the inflammatory reaction with submucosal/adventitial edema, mucus secretion, mononuclear infiltration and epithelial cell necrosis leads to obstruction of some small airways with subsequent hyperinflation, atelectasis, and wheezing. Marked airways obstruction increases respiratory effort and intrapleural negative pressure and leads to air leak occurrence. Surprisingly, the reported incidence rate of spontaneous air leaks in children is insignificant when compared with bronchiolitis prevalence [10-12].

Our review of the English literature found 13 cases of spontaneous air leaks associated with bronchiolitis (Table 1) [6-15]. Combining our case with the previously reported cases, 60% (6/10) were previously healthy. Our patient has a slight pulmonary valve stenosis, which is not a recognized risk factor for severe bronchiolitis, as it is considered a hemodynamically insignificant heart disease without pulmonary hyperflow or hypertension [5]. Four of them had history of prematurity without CLDI. Younger age does not seem to be a significant risk factor, as none was younger than 3 months. The most common air leak reported was the PNO occurring in 10/14 cases, followed by SE in 6/14 and pneumomediastinum in 3/14 children. The patients often had more than one type of air leak. In 76% (10/13), the air leak appeared in the first 4 days of the course of the disease. In our case, despite the initial clinical improvement, on the seventh day of hospitalization, the patient developed spontaneous PNO with SE. The latest spontaneous PNO in the course of bronchiolitis was described by Tutor et al [9], on the 10th day. The possible explanation for the late appearance of this complication is that medical improvement with clearing of the respiratory bronchioles, which takes several days to be achieved, “the mucociliar escalator”, could lead some inflammatory elements moving towards the upper airways, causing obstruction and increasing the negative pressure in that area and finally leading to air leakage [12]. As RSV is the most common agent of acute bronchiolitis, it is not surprising that it was the most frequent virus reported (8/12) in our review. Other reported virus included the parainfluenza, influenza A and bocavirus. This suggests that air leaks are not pathogen specific. The management of air leaks was variable. Additionally to supplemental oxygen, our patient was managed with placement of a chest drain in aspiration, because of the large PNO with important respiratory distress. Only 21% (3/14) reported patients needed mechanical ventilation.

**Table 1:** Summary of Reported Cases of Spontaneous Air Leaks Associated With Acute Bronchiolitis/Lower Viral Respiratory Infection in Children

Author	Case No.	Age (months)	Sex	Past medical history	Type of air leak	Time of presentation (days)	Pathogen	Management
Lipinski and Goodman [6]	1	8	F	Not reported	PNO	3	Not reported	Chest drain
Pollack [7]	1	4	F	Irrelevant	PNO + PNM	1	RSV	Needle aspiration
Hopkins et al [8]	1	14	M	Irrelevant	SE	2	RSV	Conservative
Tutor et al [9]	1	9	F	Irrelevant	PNM + SE	10	Influenza A	Conservative
Alter [10]	1	4	M	Not reported	PNO	2	Not reported	Ventilation, chest drain
Piastra et al [11]	2	6	F	Not reported	PNO + SE	2	RSV	Conservative
		11	F	Ex-28/40 (no CLDI)	PNO	42465	RSV	Conservative
Kambouri et al [12]	1	5	F	Not reported	PNO	7	RSV	Needle aspiration
Given et al [13]	3	10	M	Ex-PT 30/40 (no CLDI)	PNO	4	Parainfluenza 3	Conservative
		5	F	Irrelevant	PNO	3	Not identified	Conservative
		24	M	Irrelevant	PNM + SE	3	RSV	Conservative
Ursic et al [14]	1	20	F	Ex-PT 27/40 (no CLDI)	SE	Not reported	Bocavirus	Ventilation
Odek et al [15]	1	8	F	Irrelevant	PNO	3	RSV	Chest drain, ventilation
Actual case	1	21	F	Ex-PT 35/40 (no CLDI) Slight pulmonary valve stenosis	PNO + SE	7	RSV	Chest drain

PNM: pneumomediastinum; PNO: pneumothorax; PT: preterm; RSV: respiratory syncytial virus; SE: subcutaneous emphysema.

Currently, there is no recognizable treatment that can effectively interfere with the underlying pathology of bronchiolitis so as to prevent the development of air leaks [1, 3, 12]. However, once occurred, they constitute an emergency condition that demands prompt diagnosis and treatment. Physicians, especially those who work with small children must be aware for this uncommon complication that can be present late in the course of the disease despite an initial clinical improvement.
